# Supplementary material for: Dominance in self-compatibility between subgenomes of allopolyploid Arabidopsis kamchatica shown by transgenic restoration of self-incompatibility
Source: Nat Commun. 2023 Nov 29;14:7618. doi: 10.1038/s41467-023-43275-2 (PMC10687001; doi:10.1038/s41467-023-43275-2)
Supplement: Supplementary file 1 — Supplementary Information [file 41467_2023_43275_MOESM1_ESM.pdf]

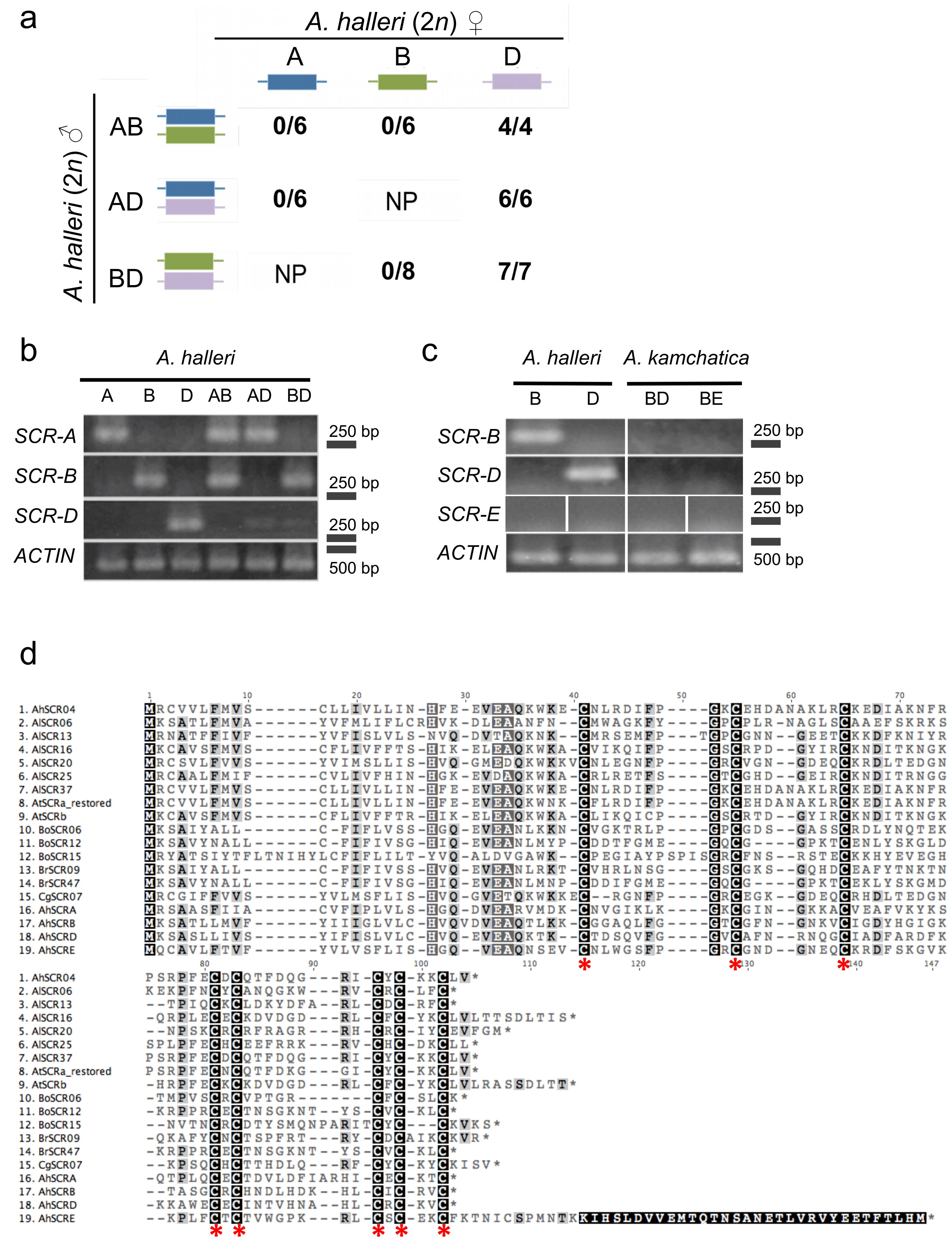

Supplementary Fig. 1.

Supplementary Fig. 1. Dominance and expression of *SCR* genes in the anther cDNAs of *Arabidopsis halleri* and *A. kamchatica* bearing different *S*-haplogroups and the alignment of amino acid sequences. **a.** Crossings were performed to investigate the dominance hierarchy of the *S*-haplogroups A, B, and D. Numerators denote crosses in which more than 20 pollen tubes penetrated the stigma, indicating a compatible reaction. Denominators denote the total number of crosses conducted in each combination. NP: not performed. See Supplementary Table 8 for the genotypes of *A. halleri* individuals. **b.** In the heterozygote of haplogroups A and B (shown as AB) of *A. halleri*, both *SCR-A* and *SCR-B* were expressed, indicating that they are co-dominant. In AD, only *SCR-A* was highly expressed, showing that *SCR-D* was recessive to *SCR-A*. Similarly, in BD, only *SCR-B* was highly expressed, showing that *SCR-D* was recessive to *SCR-B*. *ACTIN* was amplified as a quantitative control. **c.** *SCR-B* of *A. kamchatica* bearing a large insertion was not expressed in the individuals bearing haplogroups BD or BE. **d.** Alignments of amino acid sequences of 15 known SCR were used for the isolation of SCR (1–15) and the SCR of *A. halleri* isolated in this study (16–19). Ah: *A. halleri*; Al: *A. lyrata*; At: *A. thaliana*; Bo: *Brassica oleracea*; Br: *Brassica rapa*; Cg: *Capsella grandiflora*. Red asterisks indicate the eight conserved cysteine residues.

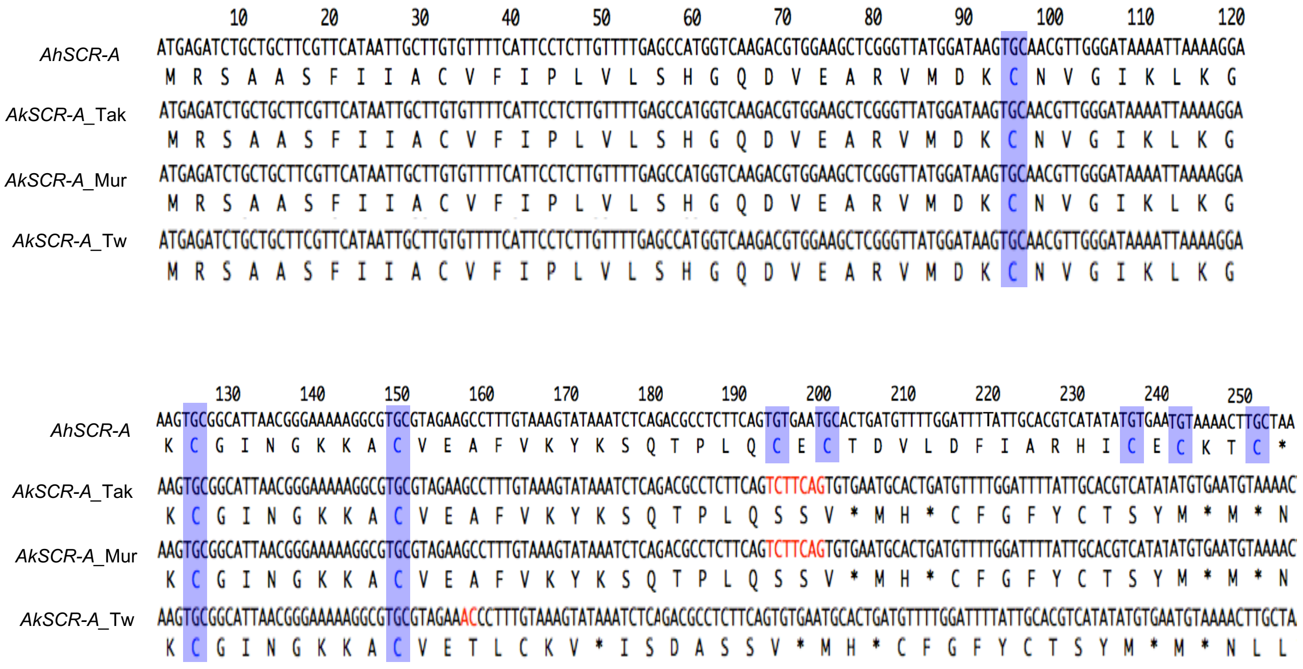

Supplementary Fig. 2. Two types of gene-disrupting mutations identified in *AkSCR-A* of *Arabidopsis kamchatica*. Alignments of DNA and amino acid sequences of *SCR-A* of *A. halleri* (*AhSCR-A*) and *A. kamchatica* samples from Takashima, Japan (*AkSCR-A*_Tak), Murodo, Japan (*AkSCR-A*_Mur) and Taiwan (*AkSCR-A*_Tw). Red: mutations in *A. kamchatica*. Blue: conserved cysteine residues.

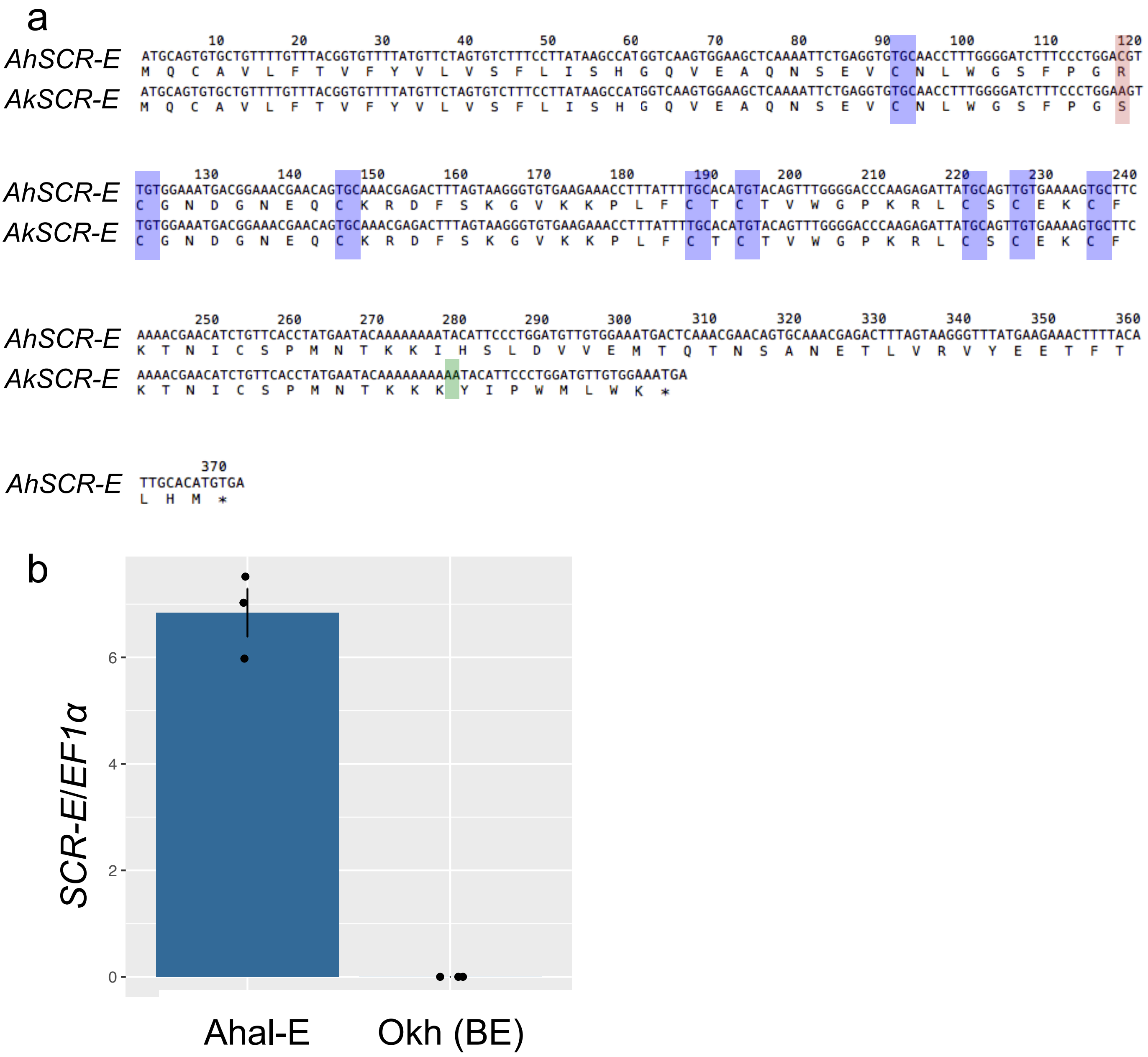

Supplementary Fig. 3. Sequence alignments of DNA and amino acid sequences of *SCR-E* of *Arabidopsis halleri* (*AhSCR-E*) and *A. kamchatica* from Okhotsk, Russia (*AkSCR-E*) and their gene expression. a. *AkSCR-E* was inherited from *A. lyrata*; thus, a divergence from *AhSCR-E* of *A. halleri* would be expected. A nucleotide substitution from cytosine to adenine resulted in the substitution of amino acids from arginine (R) to serine (S) (highlighted in red). *AhSCR-E* encoded a stretch of amino acids after the conserved eight cysteine residues (highlighted in blue), which is much longer than other *SCR* sequences (Supplementary Fig. 1d). An indel of AA (highlighted in green) was responsible for the length difference of 22 amino acids at the C-terminal. The eight cysteine residues were conserved (highlighted in blue). **b.** Expression of *AkSCR-E* in Okh (BE haplogroups) in *A. kamchatica* was very low, in contrast to that of *AhSCR-E* in *A. halleri* (mean ± S.D., $n_{\text{each line}}= 3$).

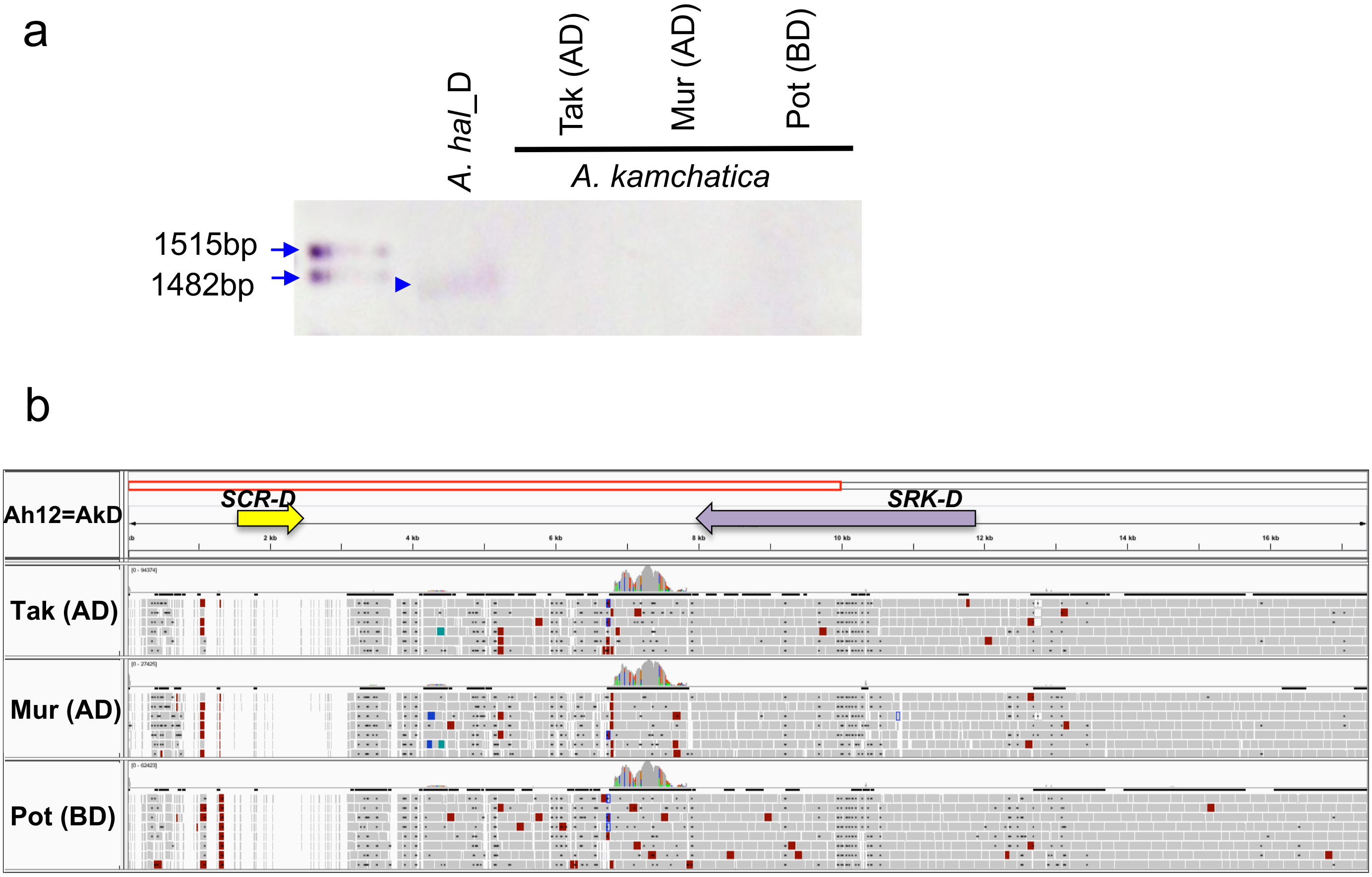

Supplementary Fig. 4. Deletion of *SCR-D* in *Arabidopsis kamchatica* bearing haplogroup D. a. Southern blotting analysis was used to examine the presence of *SCR-D* in *A. kamchatica*. Genomic DNA of *A. halleri* bearing haplogroup D (*A. hal*_D) and *A. kamchatica* samples from Takashima, Japan (Tak), Murodo, Japan (Mur), and Potter, Alaska (Pot) was digested by *Xba*I. A band was detected for *A. halleri* bearing haplogroup D (arrowhead), but no band was observed for the three samples of *A. kamchatica*, suggesting the deletion of *SCR-D* in *A. kamchatica.* A similar result was obtained from one more independent experiment. **b**. Mapping of next-generation sequencing reads of Takashima (Tak), Murodo (Mur), and Potter (Pot) accessions to the BAC sequences of the *S*-locus region of *S*-haplogroup Ah12 (=AkD) (Genbank KJ772374.1) revealed the deletion of *SCR-D* in *A. kamchatica*, where the *SCR-D* region was sparsely mapped. The locations of the *SCR-D* (<1543..>2413) and *SRK-D* (<7972..11899) genes corresponding to predicted coding sequences with introns on the BAC sequences are indicated by yellow and purple arrows, respectively. The direction of the arrow indicates the gene direction.

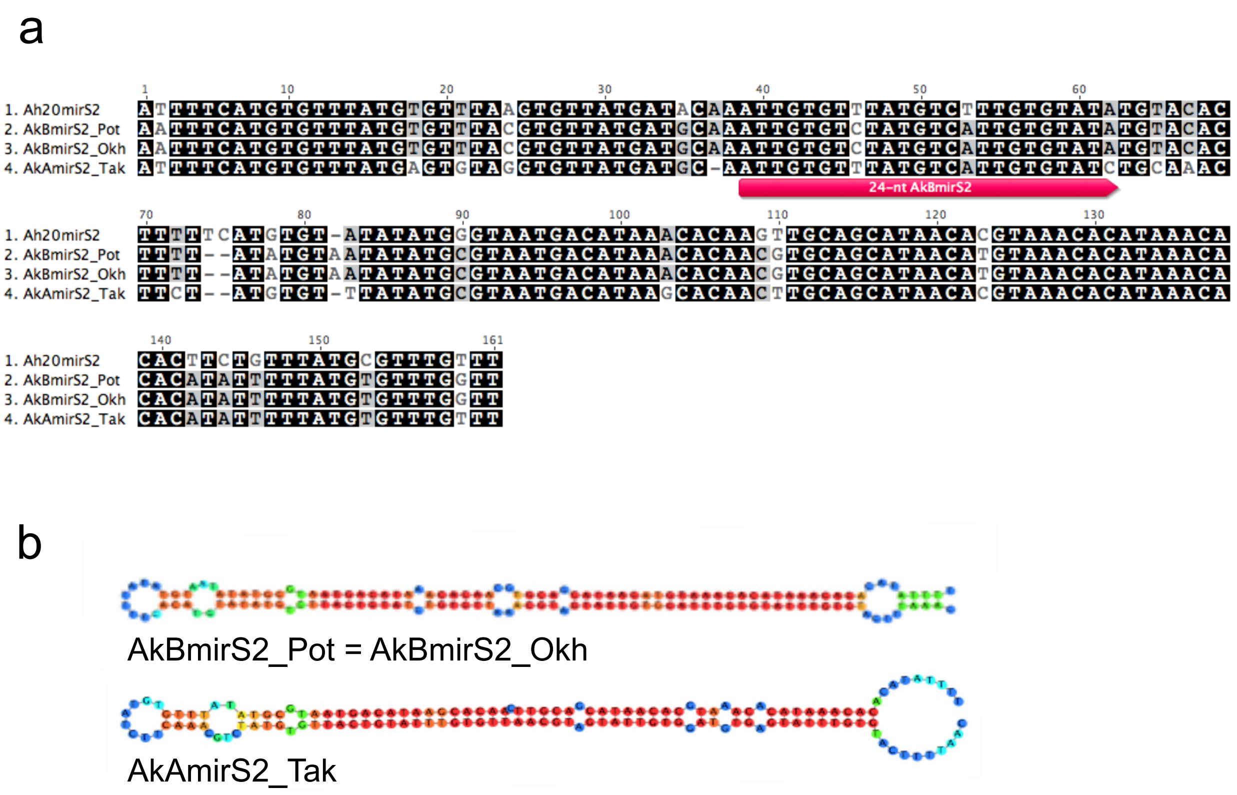

Supplementary Fig. 5. Isolation of *mirS2* family precursor genes in *A. kamchatica.* a. Precursor genes of *AkBmirS2* of *A. kamchatica* from Potter (AkBmirS2_Pot) and Okhotsk (AkBmirS2_Okh) bearing haplogroup B and *AkAmirS2* of Takashima accession bearing haplogroup A (AkAmirS2_Tak) share high sequence homology with *Ah20mirS2*. Therefore, they were classified into the *mirS2* family. Red arrow indicates the 24-nt sRNA processed from the *AkBmirS2* precursor gene, but not from *AkAmirS2*. **b**. Precursor genes of *AkBmirS2* of Potter and Okhotsk and *AkAmirS2* of Takashima accessions form imperfect stem-loop structures.

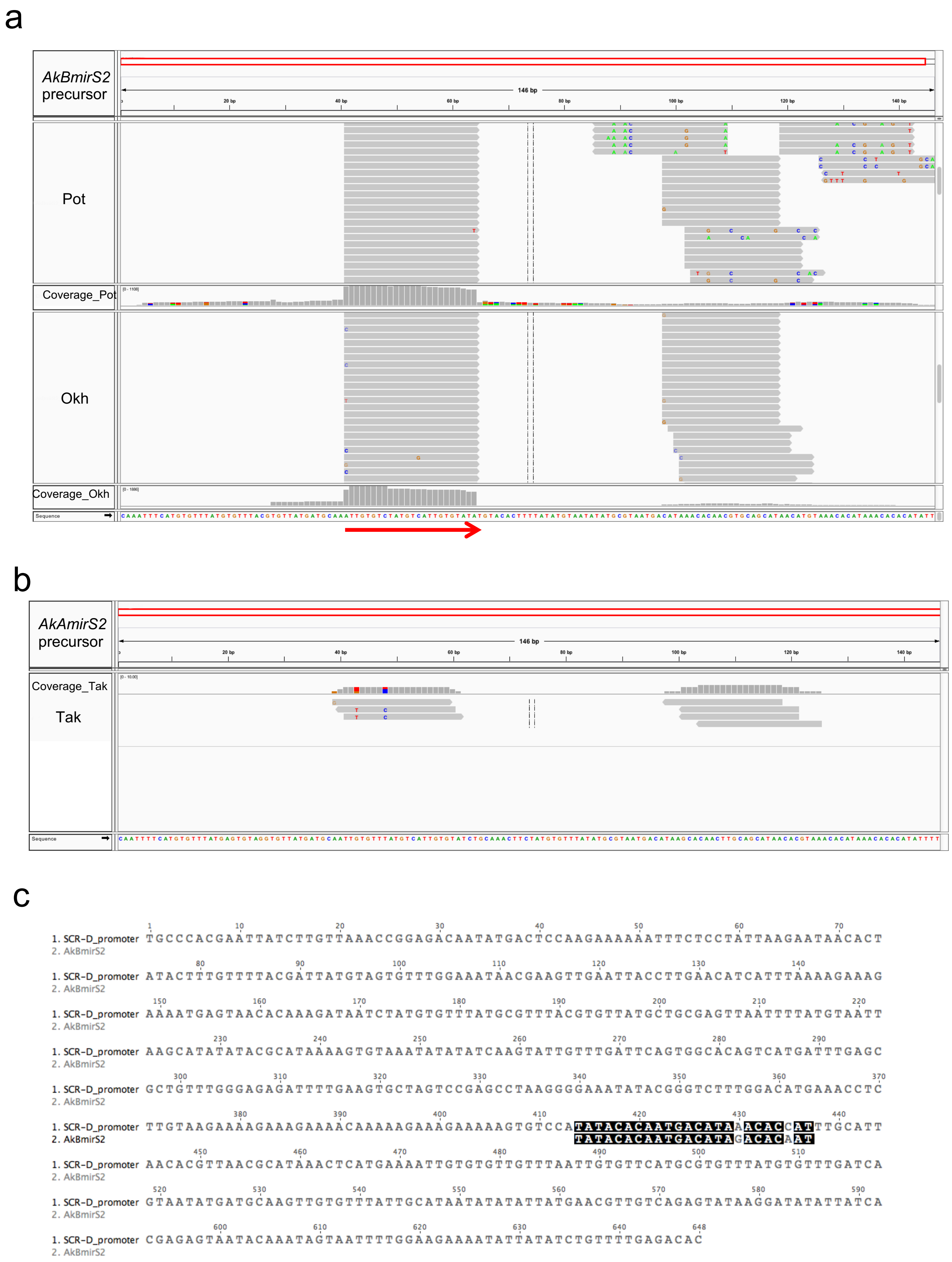

Supplementary Fig. 6

Supplementary Fig. 6. Mapping of sRNA reads of *A. kamchatica* to *mirS2* precursor genes to detect the presence of sRNA. a. sRNA reads of Potter (Pot) and Okhotsk (Okh) mapped to the *AkBmirS2* precursor gene revealed an abundance of 24-nt *AkBmirS2* showing high sequence homology with a target site at the recessive *SCR-D* promoter (red arrow). **b**. No 24nt sRNA of Takashima accession (Tak) was mapped to the *AkAmirS2* precursor gene with a 100% match. A very low number of sRNAs were mapped, but they had one or two mismatches. Identical sequences are highlighted in gray, whereas colored alphabets indicate mismatches. **c**. Potential target site of *AkBmirS2* at the promoter of the recessive *SCR-D* obtained from *A. halleri*. For visibility, the sRNA is shown as DNA in the same strand as *SCR-D*. In total, 22 of the 24 nucleotides are identical between the *AkBmirS2* sRNA and its target site.

a

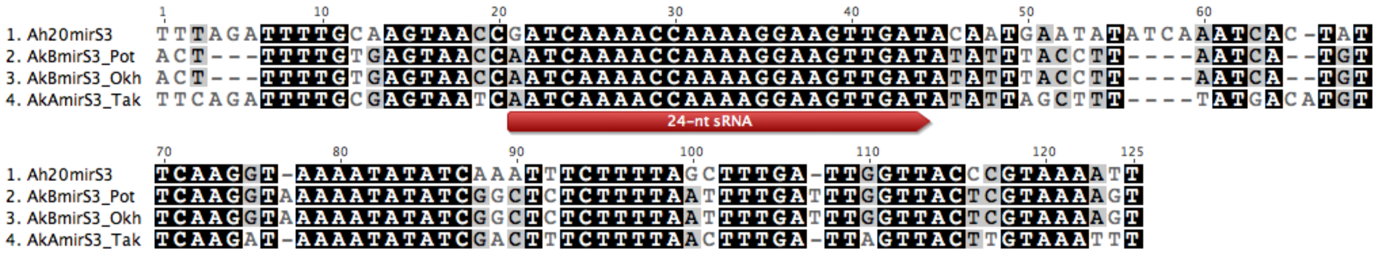

b

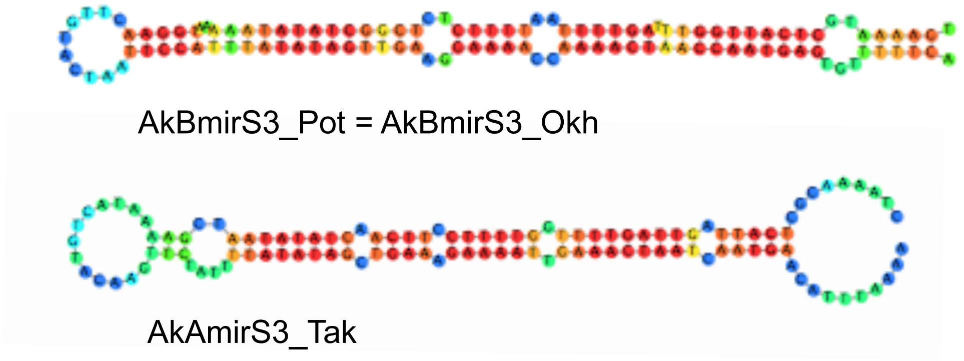

Supplementary Fig. 7. Isolation of *mirS3* family precursor genes in *A. kamchatica.* a. Precursor genes of *AkBmirS3* of *A. kamchatica* from Potter (AkBmirS3_Pot) and Okhotsk (AkBmirS3_Okh) bearing haplogroup B and *AkAmirS3* of Takashima accession bearing haplogroup A (AkAmirS3_Tak) share high sequence homology with *Ah20mirS3*. Therefore, they were classified into the *mirS3* family. The red arrow indicates the 24-nt sRNA processed from *AkBmirS2* and *AkAmirS3* precursor genes. **b**. Precursor genes of *AkBmirS3* of Potter and Okhotsk and *AkAmirS3* of Takashima accessions form imperfect stem-loop structures.

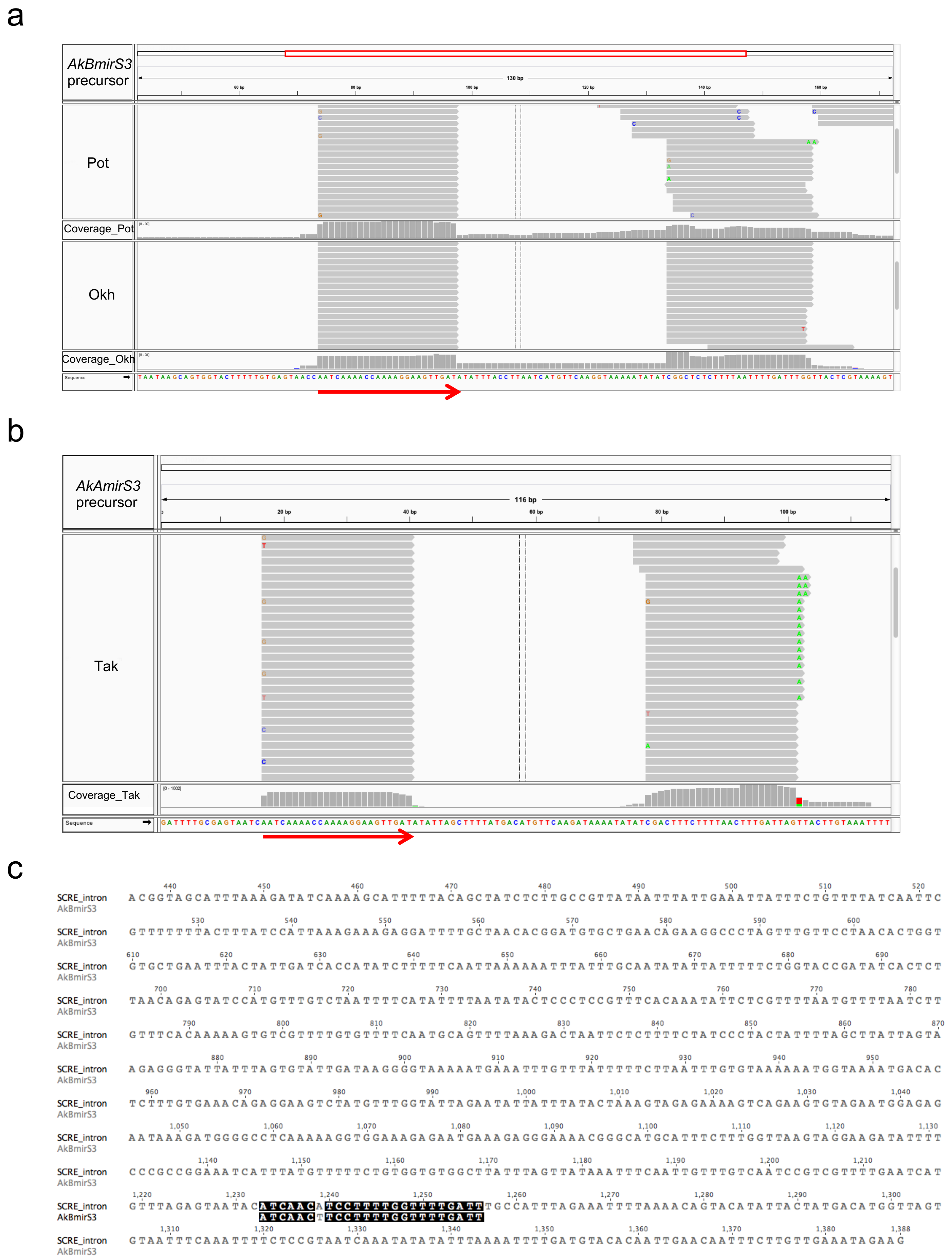

Supplementary Fig. 8.

Supplementary Fig. 8. **Mapping of sRNA reads of *A. kamchatica* to *mirS3* precursor genes to detect the presence of sRNA.** (**a**) sRNA reads of Potter (Pot) and Okhotsk (Okh) mapped to the *AkBmirS3* precursor gene, and (**b**) Takashima accession (Tak) mapped to the *AkAmirS3* precursor gene revealed the presence of 24-nt *AkBmirS3* and *AkAmirS3* (identical sequences) that show high sequence similarity with a target site at the intron of recessive *SCR-E* (red arrows). Identical sequences are highlighted in gray, whereas colored alphabets indicate mismatches. **c**. The sequence alignment of the *AkBmirS3* sRNA from the dominant *S*-haplogroup B and its potential target site at the intron of recessive *SCR-E*. For visibility, sRNA is shown as DNA in the same strand as *SCR-E*. In total, 23 of the 24 nucleotides are identical between the *AkBmirS3* sRNA and its target site.

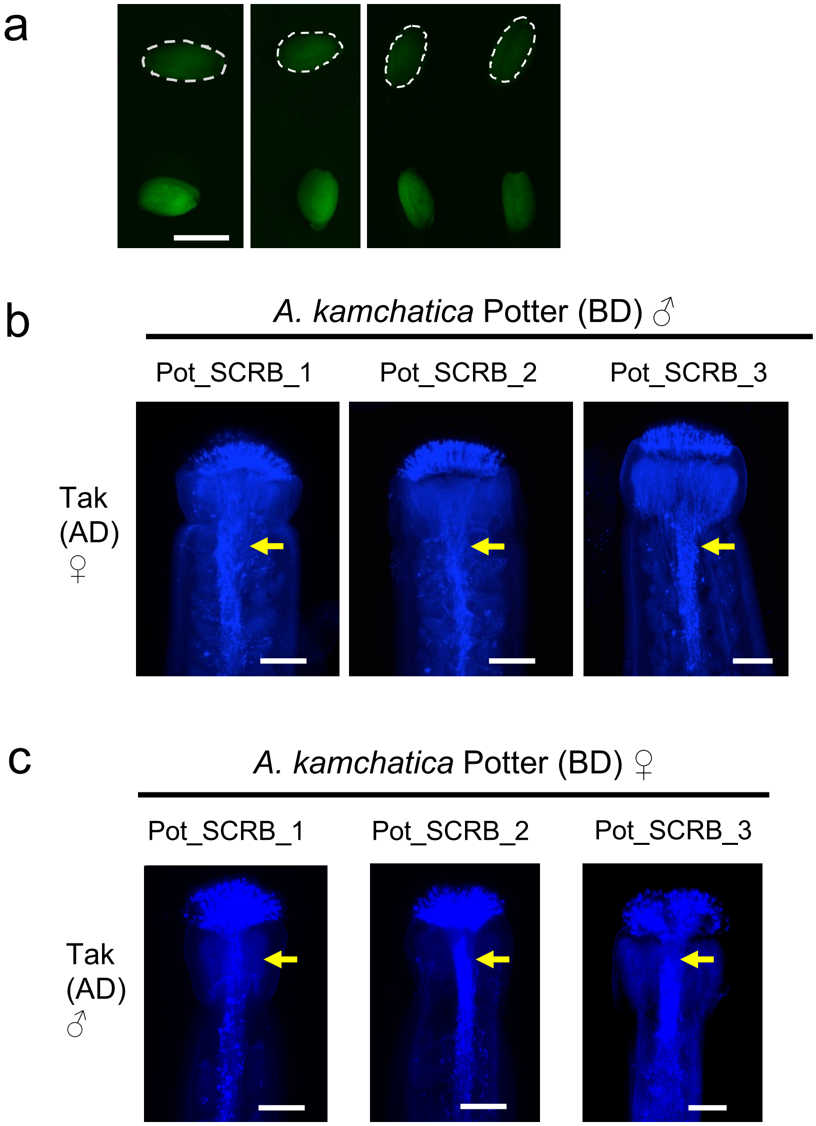

Supplementary Fig. 9. Transformation of restored *AkSCR-B* into *A. kamchatica* (Potter accessions) using the floral dip method without affecting the viability of pollen and pistils of transgenic Potter lines. a. Green fluorescent proteins expressed in the oil body membranes of seeds allowed the selection of four transformed seeds under a fluorescence stereomicroscope. Non-transformed seeds are outlined in dotted lines. **b**. Pollination of transgenic Potter with stigmas of Takashima accession (Tak) that do not bear haplogroup B confirmed pollen viability (Supplementary Table 3). **c**. Pistil viability of transgenic Potter was confirmed by pollinating pollens of Takashima accession (Tak) with their stigmas (Supplementary Table 4). *S*- haplogroup D is not functional because of the deletion of *SCR-D*. Six observations showed similar results for each crossing (sample numbers in Supplementary Tables 3 and 4). Yellow arrows indicate a bundle of pollen tubes. Scale bar = 0.25 mm.

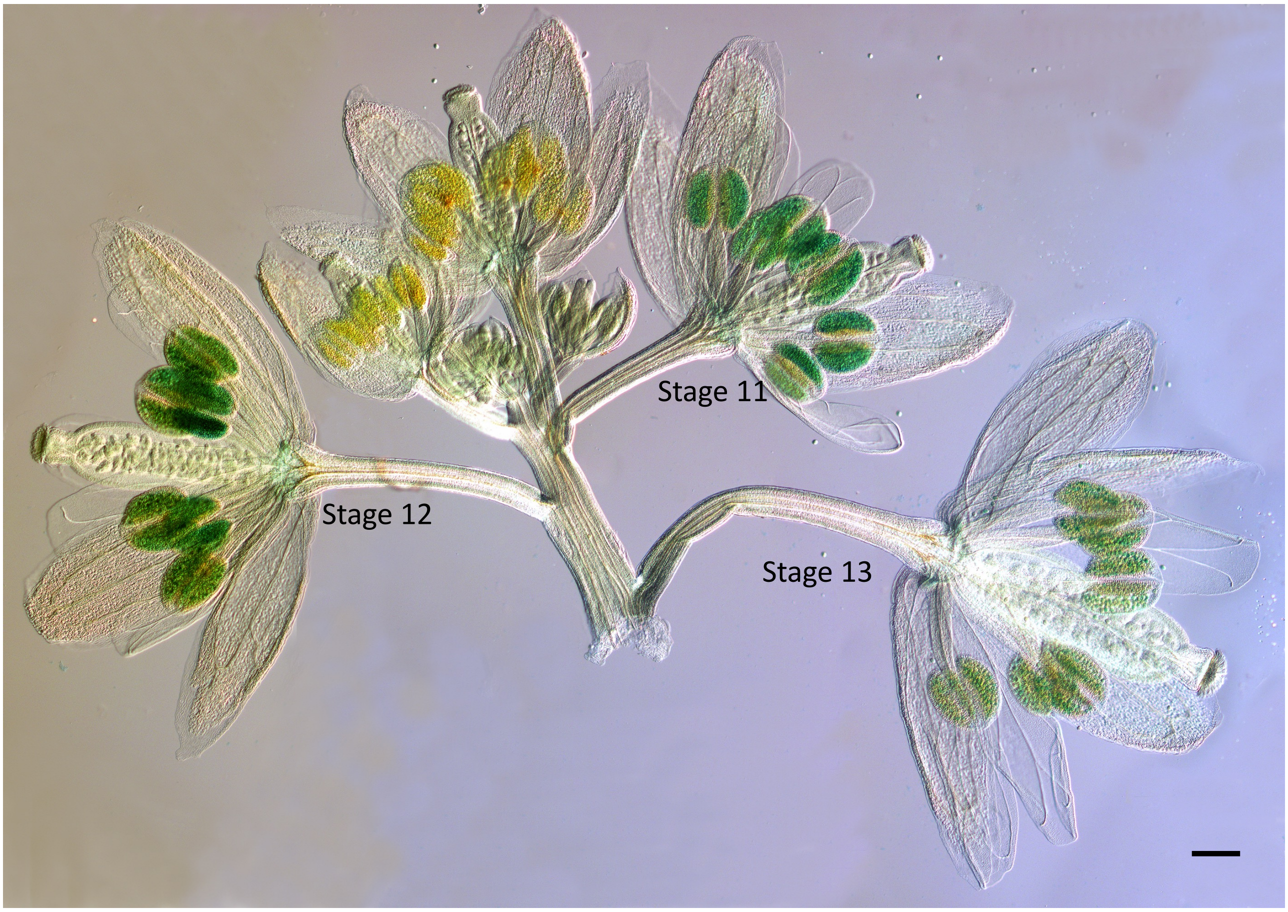

Supplementary Fig. 10. Transformation of *pAhSCR-D:GUS* into *A. thaliana.* The picture shows the expression of the promoter of *SCR-D* in pollen, in which GUS accumulation was detected more strongly in anthers at stages 11 and 12 than in anthers at stage 13 (anthesis) and younger anthers. Scale bar = 0.25 mm.

Supplementary Table 1 Segregation of *S*-locus genes in an F2 population (94 individuals) that was generated by crossing *A. kamchatica* bearing *S*-haplogroups A and D, and *A. kamchatica* bearing *S*-haplogroups B and E. Results of PCR-based genotyping are shown. "y" indicates amplification.

DNA Serial Num	F1 name	F2 name	*SRK-A*	*SCR-A*	*AkAmirS2*	*SRK-B*	*SCR-B*	*AkBmirS2*	*AkBmirS3*	*SRK-E*	*SCR-E*
1	W1892-8	1	y	y	y	y	y	y	y	y	y
2	W1892-8	2	y	y	y	y	y	y	y	y	y
3	W1892-8	3	y	y	y	y	y	y	y	y	y
4	W1892-8	4	y	y	y	y	y	y	y	y	y
5	W1892-8	5	y	y	y					y	y
6	W1892-8	6				y	y	y	y	y	y
7	W1892-8	7	y	y	y	y	y	y	y	y	y
8	W1892-8	8	y	y	y					y	y
9	W1892-8	9	y	y	y					y	y
10	W1892-8	10	y	y	y					y	y
11	W1892-8	11	y	y	y	y	y	y	y	y	y
12	W1892-8	12	y	y	y	y	y	y	y	y	y
13	W1892-8	13	y	y	y					y	y
14	W1892-8	14				y	y	y	y	y	y
15	W1892-8	15				y	y	y	y	y	y
16	W1892-8	16	y	y	y					y	y
17	W1892-8	17	y	y	y	y	y	y	y	y	y
18	W1892-8	18	y	y	y	y	y	y	y	y	y
19	W1892-8	19				y	y	y	y	y	y
20	W1892-8	20	y	y	y	y	y	y	y	y	y
21	W1892-8	21	y	y	y	y	y	y	y	y	y
22	W1892-8	22	y	y	y	y	y	y	y	y	y
23	W1892-8	23	y	y	y					y	y
24	W1892-8	24	y	y	y	y	y	y	y	y	y
25	W1892-8	25	y	y	y					y	y
26	W1892-8	26	y	y	y	y	y	y	y	y	y
27	W1892-8	27	y	y	y	y	y	y	y	y	y
28	W1892-8	28	y	y	y	y	y	y	y	y	y
29	W1892-8	29	y	y	y						
30	W1892-8	30	y	y	y	y	y	y	y		
31	W1892-8	31	y	y	y					y	y
32	W1892-8	32				y	y	y	y	y	y
33	W1892-8	33	y	y	y					y	y
34	W1892-8	34	y	y	y						
35	W1892-8	35	y	y	y					y	y
36	W1892-8	36	y	y	y					y	y
37	W1892-8	37				y	y	y	y	y	y
38	W1892-8	38				y	y	y	y	y	y
39	W1892-8	39	y	y	y	y	y	y	y	y	y
40	W1892-8	40	y	y	y	y	y	y	y	y	y
41	W1892-8	41	y	y	y					y	y
42	W1892-8	42	y	y	y	y	y	y	y	y	y
43	W1892-8	43				y	y	y	y	y	y
44	W1892-8	44	y	y	y	y	y	y	y	y	y
45	W1892-8	45	y	y	y					y	y
46	W1892-8	46	y	y	y	y	y	y	y	y	y
47	W1892-8	47				y	y	y	y	y	y
48	W1892-8	48	y	y	y	y	y	y	y	y	y
49	W1892-8	49	y	y	y	y	y	y	y	y	y
50	W1892-8	50	y	y	y	y	y	y	y	y	y
51	W1892-8	51	y	y	y	y	y	y	y	y	y
52	W1892-8	52	y	y	y					y	y
53	W1892-8	53	y	y	y					y	y
54	W1892-8	54	y	y	y	y	y	y	y	y	y
55	W1892-8	55	y	y	y	y	y	y	y	y	y
56	W1892-8	56	y	y	y	y	y	y	y	y	y
57	W1892-8	57				y	y	y	y	y	y
58	W1892-8	58	y	y	y	y	y	y	y		
59	W1892-8	59	y	y	y	y	y	y	y	y	y
60	W1892-8	60	y	y	y	y	y	y	y	y	y
61	W1892-8	61	y	y	y	y	y	y	y	y	y
62	W1892-8	62	y	y	y	y	y	y	y	y	y
63	W1892-8	63	y	y	y					y	y
64	W1892-8	64	y	y	y					y	y
65	W1892-8	65	y	y	y	y	y	y	y	y	y
66	W1892-8	66	y	y	y	y	y	y	y	y	y
67	W1892-8	67	y	y	y					y	y
68	W1892-8	68	y	y	y	y	y	y	y		
69	W1892-8	69	y	y	y	y	y	y	y	y	y
70	W1892-8	70	y	y	y	y	y	y	y	y	y
71	W1892-8	71				y	y	y	y	y	y
72	W1892-8	72				y	y	y	y	y	y
73	W1892-8	73	y	y	y	y	y	y	y	y	y
74	W1892-8	74	y	y	y	y	y	y	y		
75	W1892-8	75				y	y	y	y		
76	W1892-8	76				y	y	y	y	y	y
77	W1892-8	77				y	y	y	y	y	y
78	W1892-8	78	y	y	y	y	y	y	y	y	y
79	W1892-8	79	y	y	y	y	y	y	y	y	y
80	W1892-8	80	y	y	y					y	y
81	W1892-8	81	y	y	y						
82	W1892-8	82	y	y	y	y	y	y	y	y	y
83	W1892-8	83				y	y	y	y	y	y
84	W1892-8	84	y	y	y	y	y	y	y	y	y
85	W1892-8	85	y	y	y	y	y	y	y	y	y
86	W1892-8	86	y	y	y	y	y	y	y	y	y
87	W1892-8	87	y	y	y	y	y	y	y	y	y
88	W1892-8	88	y	y	y	y	y	y	y	y	y
89	W1892-8	89	y	y	y					y	y
90	W1892-8	90	y	y	y	y	y	y	y	y	y
91	W1892-8	91	y	y	y					y	y
92	W1892-8	92	y	y	y					y	y
93	W1892-8	93	y	y	y	y	y	y	y		
94	W1892-8	94	y	y	y						

Supplementary Table 2 Summary of results of self-pollination of transgenic Potter transformed with restored *AkSCR-B* (Pot_SCRB).

		Self-pollination	
		<20 pollen tubes	>=20 pollen tubes
Transgenic lines	Pot_SCRB_1	10	0
	Pot_SCRB_2	6	0
	Pot_SCRB_3	5	0
	Pot_SCRB_4	0	5

<20 pollen tubes: Penetration of stigma by <20 pollen tubes, suggesting an incompatible reaction

>=20 pollen tubes : Penetration of stigma by >=20 pollen tubes, suggesting a compatible reaction

Supplementary Table 3 Summary of results of crossings between pollen of transgenic Potter transformed with restored *AkSCR-B* (Pot_SCRB) and stigmas of Takashima accession bearing haplogroup A and D (Tak) to check the viability of transgenic pollen.

		Tak (stigma)	
		<20 pollen tubes	>=20 pollen tubes
Transgenic lines (pollen)	Pot_SCRB_1	0	6
	Pot_SCRB_2	0	6
	Pot_SCRB_3	0	6

<20 pollen tubes: Penetration of stigma by <20 pollen tubes, suggesting an incompatible reaction

>=20 pollen tubes : Penetration of stigma by >=20 pollen tubes, suggesting a compatible reaction

Supplementary Table 4 Summary of results of crossings between pollen of Takashima accession (Tak) and stigmas of transformed with restored *AkSCR-B* (Pot_SCRB) to check the viability of transgenic pistil.

		Tak (Pollen)	
		<20 pollen tubes	>=20 pollen tubes
Transgenic lines (stigma)	Pot_SCRB_1	0	6
	Pot_SCRB_2	0	6
	Pot_SCRB_3	0	6

<20 pollen tubes: Penetration of stigma by <20 pollen tubes, suggesting an incompatible reaction

>=20 pollen tubes : Penetration of stigma by >=20 pollen tubes, suggesting a compatible reaction

Supplementary Table 5 Summary of results of self-pollination of transgenic Potter transformed with *AhSCR-D* (Pot_SCRD).

		Self-pollination	
		<20 pollen tubes	>=20 pollen tubes
Transgenic lines	Pot_SCRD_1	0	6
	Pot_SCRD_3	0	6
	Pot_SCRD_4	0	6
	Pot_SCRD_6	0	6

<20 pollen tubes: Penetration of stigma by <20 pollen tubes, suggesting an incompatible reaction

>=20 pollen tubes : Penetration of stigma by >=20 pollen tubes, suggesting a compatible reaction

Supplementary Table 6 Summary of results of self-pollination of transgenic *A. kamchatica* Takashima accession transformed with *pAhSCRD:AhSCRD*.

		Self-pollination	
		<20 pollen tubes	>=20 pollen tubes
Transgenic lines	Tak_pAhSCRD:AhSCRD_7	5	1
	Tak_pAhSCRD:AhSCRD_8	6	1
	Tak_pAhSCRD:AhSCRD_9	2	4
	Tak_pAhSCRD:AhSCRD_10	3	3
	Tak_pAhSCRD:AhSCRD_11	4	2
	Tak_pAhSCRD:AhSCRD_12	5	1
	Tak_pAhSCRD:AhSCRD_13	6	0

<20 pollen tubes: Penetration of stigma by <20 pollen tubes, suggesting an incompatible reaction

>=20 pollen tubes : Penetration of stigma by >=20 pollen tubes, suggesting a compatible reaction

Supplementary Table 7 P-values**.** P-values were calculated by two-sided tests with adjustments for multiple comparisons (post hoc Tukey test)

P-values for Fig 2	length of silique	seed number
WT vs. Pot_SCRB_1	4.71E-12	2.37E-09
WT vs. Pot_SCRB_2	6.48E-13	1.74E-09
WT vs. Pot_SCRB_3	1.57E-12	1.49E-09
WT vs. Pot_SCRB_4	5.46E-01	5.35E-01

P-values for Fig 3	length of silique	seed number
WT vs. Pot_SCRD_1	0.99	0.94
WT vs. Pot_SCRD_3	0.32	0.33
WT vs. Pot_SCRD_4	0.64	0.17
WT vs. Pot_SCRD_6	0.77	0.71

P-values for Fig 4	length of silique	seed number
WT vs. SCRD_7	1.39E-05	6.55E-07
WT vs. SCRD_8	1.21E-08	1.83E-09
WT vs. SCRD_9	5.32E-02	3.64E-03
WT vs. SCRD_10	2.76E-04	1.37E-05
WT vs. SCRD_11	1.40E-02	2.52E-04
WT vs. SCRD_12	2.45E-06	1.15E-07
WT vs. SCRD_13	4.67E-09	9.75E-10

Supplementary Table 8 Plant materials used in this study

	**Species**	Area	**Population**	***S*-haplogroups**
1	*Arabidopsis kamchatica*	Mountains in Central Honshu, Japan	Japan, Toyama, Mt. Shirouma	B, D
2	*Arabidopsis kamchatica*	Hokkaido, Japan	Japan, Hokkaido, Asahikawa, Sounkyo	B, D
3	*Arabidopsis kamchatica*	Far East Russia	Russia, Kamchatskii krai, Nachiki, basin of the Nachikinskoe ozero lake	B, D
4	*Arabidopsis kamchatica*	Far East Russia	Russia, Kamchatskii krai, near the road from Petropavlovsk Kamchatskii to Esso	B, D
5	*Arabidopsis kamchatica*	Far East Russia	Russia, Kamchatskii krai, close to the bridge over the river Denokhonok	B, D
6	*Arabidopsis kamchatica*	Far East Russia	Russia, Kamchatskii krai, Petropavlovsk Kamchatskii, Mishenaya gora	B, C, D
7	*Arabidopsis kamchatica*	Far East Russia	Russia, Kamchatskii krai, Elizovo	B, D
8	*Arabidopsis kamchatica*	Far East Russia	Russia, Kamchatskii krai, Nachiki, Mt. Nachikinskoe zerkaltse	B, E
9	*Arabidopsis kamchatica*	Far East Russia	Russia, Kamchatskii krai, Petropavlovsk Kamchatskii, Avachinskaya sopka	B, D
10	*Arabidopsis kamchatica*	Far East Russia	Russia, Kamchatskii krai, close to the bridge over the river Pravaya Kamchatka	B, D
11	*Arabidopsis kamchatica*	Far East Russia	Russia, Khabarovsk krai, Okhotskii raion, foothills of the Lanzhinskie gory Mountains (Okhotsk)	B, E
12	*Arabidopsis kamchatica*	Far East Russia	Russia, Kamchatskii krai, Esso, Srednii kamchatskii khrebe	B, E
13	*Arabidopsis kamchatica*	Alaska	USA, Alaska, Kenai	B, D
14	*Arabidopsis kamchatica*	Alaska	USA, Alaska, Chugach State Park, Potter (Potter)	B, D
15	*Arabidopsis kamchatica*	Alaska	USA, Alaska, Healy	B, D
16	*Arabidopsis kamchatica*	Alaska	USA, Alaska, Chena River, Chena Hot Springs Rd.	B, D
17	*Arabidopsis kamchatica*	Alaska	USA, Alaska, Richardson Highway, South of Darling Creek bridge	B, D
18	*Arabidopsis kamchatica*	Alaska	USA, Alaska, Portage Bay Rd	B, D
19	*Arabidopsis kamchatica*	Canada and Washington	Canada, Yukon, Mush Lake	B, D
20	*Arabidopsis kamchatica*	Canada and Washington	USA, Washington, Mt. Baker	B, D
21	*Arabidopsis kamchatica*	Canada and Washington	USA, Washington, Mt. Baker	B, D
22	*Arabidopsis halleri* subsp. *gemmifera*	Lowland in Western Honshu, Japan	Japan, Osaka, Tada-Ginzan	A, X*
23	*Arabidopsis halleri* subsp. *gemmifera*	Lowland in Western Honshu, Japan	Japan, Osaka, Tada-Ginzan	B, X*
24	*Arabidopsis halleri* subsp. *gemmifera*	Lowland in Western Honshu, Japan	Japan, Shiga, Mt. Ibuki	D, C
25	*Arabidopsis halleri* subsp. *gemmifera*	Lowland in Western Honshu, Japan	Japan, Hyogo, Omoide River	E, C
26	*Arabidopsis kamchatica*	Taiwan	Taiwan, Taroko N.P., close to the entrance of the park (Taiwan)	A, D
27	*Arabidopsis kamchatica* subsp. *kawasakiana*	Lowland in Western Honshu, Japan	Japan, Shiga, Takashima (Takashima)	A, D
28	*Arabidopsis kamchatica* subsp. *kawasakiana*	Lowland in Western Honshu, Japan	Japan, Shiga, Ohtsu, Ohmimaiko	A, D
29	*Arabidopsis kamchatica* subsp. *kawasakiana*	Lowland in Western Honshu, Japan	Japan, Shiga, Hikone	A, D
30	*Arabidopsis kamchatica*	Mountains in Central Honshu, Japan	Japan, Toyama, Murodo	A, D
31	*Arabidopsis kamchatica*	Mountains in Central Honshu, Japan	Japan, Toyama, Tsurugigozen	A, D
32	*Arabidopsis kamchatica*	Mountains in Central Honshu, Japan	Japan, Toyama, Tateyama, Mikurigaike	A, D
33	*Arabidopsis kamchatica*	Mountains in Central Honshu, Japan	Japan, Toyama, Kurobe-dam	A, D
34	*Arabidopsis kamchatica*	Mountains in Central Honshu, Japan	Japan, Nagano, Kamikochi, Myojin	A, D
35	*Arabidopsis kamchatica*	Lowland in Northern Honshu, Japan	Japan, Niigata, Tsugawa	A, D

X* Unknown S-haplogroup that was phenotypically recessive

Supplementary Table 9 Primer List

Haplogroup-specific primers used for the PCR-based genotyping					Annealing Temp (°C)	Elongation time (sec)	Reference
SCR-A	SCRA_9568F2	GAAAAAGGCGTGCGTAGAAG	SCRA_9568R	TTTTTGGTCATAAGACTGTGGTT	60	30	this study
SCR-B	SCRB_4951F4	GTGGAAGCTCAAACGCTGAAG	SCRB_4951R	GTATCCGATGATATGGATGTC	60	30	this study
SCR-D	SCRD_13360F4	GATGCATAGCAGATTTTGCTAGG	SCRD_13360R	CGCATCGCAGGTAAGCCTAC	60	30	this study
SCR-E	SCRE_denovoF4	CAACCTTTGGGGATCTTTCC	SCRE_denovoR6	TTTTGTGTCCCCAACATTCA	60	30	this study
AkSRK-A	Akam13-22F1	GCAGAGTTTTGATTATCCAACAGAT	Akam13-22R1	AGTCTTTCTCATCAATTTCCATGTC	62	60	Tsuchimatsu et al. (2012)
AkSRK-B	Akam13-23F1	CGATTTTCCCACTGATACTCTACTC	Akam13-23R1	GACAACTCTTCTTGCATTCTTTTGT	62	60	Tsuchimatsu et al. (2012)
AkSRK-D	Akam13-42genF4	ATGAGAGGTTTACGAAATATCTTCCA	Akam13-42R4b	ACTGTGTTCCACTGCCATGA	60	60	Tsuchimatsu et al. (2012)
AkSRK-E	Akam13-17F15	TTCCTGGAAAAGCTCATTTG	Akam13-17R5	GAACCCACTCAAATCGCTGT	60	60	Tsuchimatsu et al. (2012)

Primers for full-length *SCR* sequences					Annealing Temp (°C)	Elongation time (sec)	Reference
SCR-A	SCRA_9568F3	CAACAAACTTCTGAGATTGCTCT	SCRA_9568R	TTTTTGGTCATAAGACTGTGGTT	60	120	this study
AhSCR-B	SCRB_4951F9	GATAAACAAAATTCCAACAAAAGC	SCRB_4951R	GTATCCGATGATATGGATGTC	60	120	this study
AkSCR-B	SCRB_4951F9	GATAAACAAAATTCCAACAAAAGC	SCRB_4951iR4	CCATGCCTTGCCTTCTTATG	60	240	this study
AkSCR-B	SCRB_4951iF2	GTTGTACTCCTAATGGGAAGTG	SCRB_4951R	GTATCCGATGATATGGATGTC	60	240	this study
SCR-D	SCRD_13360F8	CACGAGAGTAATACAAATAGTAATTTTGG	SCRD_13360R	CGCATCGCAGGTAAGCCTAC	60	180	this study
SCR-E	SCRE_denovoF5	GCTAAGGACTATCAAGCCGAGA	SCRE_denovoR6	TTTTGTGTCCCCAACATTCA	60	180	this study

Primers for RT_PCR					Annealing Temp (°C)	Elongation time (sec)	Reference
SCR-A	SCRA_9568F5	GAGTCATGAGATCTGCTGC	SCRA_9568R	TTTTTGGTCATAAGACTGTGGTT	60	30	this study
SCR-B	SCRB_4951F5	CATAGGCCTTGTTTTGTGTCAC	SCRB_4951R	GTATCCGATGATATGGATGTC	60	30	this study
SCR-D	SCRD_13360F2	GAGACACATGAAGTCTGCATC	SCRD_13360R	CGCATCGCAGGTAAGCCTAC	60	30	this study
SCR-D (Fig. 3a)	SCR42-13360F6	ATCGCTTGTTTTGTGTCACG	SCR42-13360R2	CCTAGCAAAATCTGCTATGCATC	60	60	this study
SCR-E	SCRE_denovoF5	GCTAAGGACTATCAAGCCGAGA	SCRE_denovoR5	TTCCACAACATCCAGGGAAT	60	30	this study
ACTIN	Actin8F	ATGAAGATTAAGGTCGTGGCA	Actin8R	TCCGAGTTTGAAGAGGCTAC	60	30	Miyashima et al. Plant Cell Phys 50:626, 2009
EF1α	3529_AhEF1aF	TGAGCACGCTCTTCTTGCTTTCA	3530_AhEF1aR	GGTGGTGGCATCCATCTTGTTACA	60	60	Becher et al. Plant Journal 37:251, 2004

Primers for RT-qPCR					Annealing Temp (°C)	Elongation time (sec)	Reference
SCR-B	SCR23-4951F3	CATCTTTTCGGAGGCATGAAG	SCR23-4951R3	CTTCAGCGTTTGAGCTTCCAC	60	60	this study
SCR-E	SCRE_F10	GCAACGTATAAACACATATGCTAAGG	SCRE_RT_R1	GAGCTTCCACTTGACCATGG	60	60	this study
EF1α	3529_AhEF1aF	TGAGCACGCTCTTCTTGCTTTCA	3530_AhEF1aR	GGTGGTGGCATCCATCTTGTTACA	60	60	Becher et al. Plant Journal 37:251, 2004

Primers for probe of *SCR-D* for Southern blot					Annealing Temp (°C)	Elongation time (sec)	Reference
SCR-D	SCRD_13360F11	GGTGGAAGCACAGAAGACGAAG	SCRD_13360R9	CAAAGCCAAAAAGAAATGATGTAGG	60	30	this study

Primers for full-length *SCR* sequences, including promoter regions for transformation into *A. kamchatica*					Annealing Temp (°C)	Elongation time (sec)	Reference
AkSCR-B_1°	SCRB_4951promF1	TGTTCCATGAATAGTGAGATTTTTG	SCRB_4951R8	GTTCATCCTATTTAAAACTCTTTATTTTTCTG	60	300	this study
AkSCR-B_1°	SCRB_4951F8	TAAAGAGTTTTAAATAGGATGAACATTATTAC	SCRB_4951R	GTATCCGATGATATGGATGTC	60	300	this study
AkSCR-B_2°	SCRB_4951promF1	TGTTCCATGAATAGTGAGATTTTTG	SCRB_4951R	GTATCCGATGATATGGATGTC	60	300	this study
AhSCR-D	SCRD_13360F18	TGCCCACGAATTATCTTGTT	SCRD_13360R3	AAAAGAAAATCCAGCGCATC	60	300	this study
AhSCR-D_prom	SCRD_13360F18	TGCCCACGAATTATCTTGTT	SCRD_13360R5	CGTGACACAAAACAAGCGAT	60	60	this study

Primers for validation of transformation of *SCR* genes into *A. kamchatica*					Annealing Temp (°C)	Elongation time (sec)	Reference
AkSCR-B_prom	pFAST_t35S_F	TTCGCTCATGTGTTGAGCAT	SCRB_4951promR2	TTGCACACCTTTTAAGGGGATAATCGT	60	30	this study
AkSCR-B	SCRB_4951F4	GTGGAAGCTCAAACGCTGAAG	pFAST_RB_R	AAACTGAAGGCGGGAAACGAC	60	30	this study
AhSCR-D_prom	pFAST_t35S_F	TTCGCTCATGTGTTGAGCAT	SCRD_13360R20	TTCATGTCCAAAGACCCGTA	60	30	this study
AhSCR-D	SCRD_13360F4	GATGCATAGCAGATTTTGCTAGG	pFAST_RB_R	AAACTGAAGGCGGGAAACGAC	60	30	this study

Primers for sRNA precursors					Annealing Temp (°C)	Elongation time (sec)	Reference
AkAmirS2	AkAsmiR2_F1	AACACAACTTGCACCGTAATACTC	AkAsmiR2_R1	TGTTTCAAAACATGAAGGTTGA	60	30	this study
AkBmirS2	AkBsmiR2_F1	CAACTAAACACAATACACAAATTTCA	AkBsmiR2_R1	CCTTGAACATCATTTGAAAGAAA	60	30	this study
AkBmirS3	AkBmirS3_F1	TGCTTTTCCCATGTTATTGTAA	AkBmirS3_R1	CCAAGAACCCAAAAACTCCA	60	30	this study

Supplementary Table 10 Segregation of *SCR-D* in an F2 population (32 individuals) that was generated by crossing *A. halleri* bearing haplogroup D and *A. halleri* bearing haplogroup A or B. Results of PCR-based genotyping are shown. "y" indicates amplification.

DNA Serial Num	F1 name	F2 name	*SRK-D*	*SCR-D*
1	AhalBxAhalD	1	y	y
2	AhalBxAhalD	2		
3	AhalBxAhalD	3	y	y
4	AhalBxAhalD	4		
5	AhalBxAhalD	5	y	y
6	AhalBxAhalD	6	y	y
7	AhalBxAhalD	7	y	y
8	AhalBxAhalD	8		
9	AhalBxAhalD	9	y	y
10	AhalDxAhalA	1		
11	AhalDxAhalA	2		
12	AhalDxAhalA	3	y	y
13	AhalDxAhalA	4		
14	AhalDxAhalA	5		
15	AhalDxAhalA	6	y	y
16	AhalDxAhalA	7	y	y
17	AhalDxAhalA	8		
18	AhalDxAhalA	9	y	y
19	AhalDxAhalB	1		
20	AhalDxAhalB	2		
21	AhalDxAhalB	3		
22	AhalDxAhalB	4	y	y
23	AhalDxAhalB	5	y	y
24	AhalDxAhalB	6	y	y
25	AhalDxAhalB	7	y	y
26	AhalDxAhalB	8		
27	AhalAxAhalD	1		
28	AhalAxAhalD	2		
29	AhalAxAhalD	3		
30	AhalAxAhalD	4		
31	AhalAxAhalD	5		
32	AhalAxAhalD	6	y	y
