# Supplementary material for: Dominance in self-compatibility between subgenomes of allopolyploid Arabidopsis kamchatica shown by transgenic restoration of self-incompatibility
Source: Nat Commun. 2023 Nov 29;14:7618. doi: 10.1038/s41467-023-43275-2 (PMC10687001; doi:10.1038/s41467-023-43275-2)
Supplement: Supplementary file 3 — Reporting Summary [file 41467_2023_43275_MOESM3_ESM.pdf]

nature portfolio

Corresponding author(s): Kentaro Shimizu

Last updated by author(s): Oct 12, 2023

Reporting Summary

Nature Portfolio wishes to improve the reproducibility of the work that we publish. This form provides structure for consistency and transparency in reporting. For further information on Nature Portfolio policies, see our Editorial Policies and the Editorial Policy Checklist.

Statistics

For all statistical analyses, confirm that the following items are present in the figure legend, table legend, main text, or Methods section.

n/a	Confirmed	
☐	☒	The exact sample size (*n*) for each experimental group/condition, given as a discrete number and unit of measurement
☒	☐	A statement on whether measurements were taken from distinct samples or whether the same sample was measured repeatedly
☐	☒	The statistical test(s) used AND whether they are one- or two-sided<br>*Only common tests should be described solely by name; describe more complex techniques in the Methods section.*
☒	☐	A description of all covariates tested
☐	☒	A description of any assumptions or corrections, such as tests of normality and adjustment for multiple comparisons
☐	☒	A full description of the statistical parameters including central tendency (e.g. means) or other basic estimates (e.g. regression coefficient) AND variation (e.g. standard deviation) or associated estimates of uncertainty (e.g. confidence intervals)
☐	☒	For null hypothesis testing, the test statistic (e.g. *F*, *t*, *r*) with confidence intervals, effect sizes, degrees of freedom and *P* value noted<br>*Give P values as exact values whenever suitable.*
☒	☐	For Bayesian analysis, information on the choice of priors and Markov chain Monte Carlo settings
☒	☐	For hierarchical and complex designs, identification of the appropriate level for tests and full reporting of outcomes
☒	☐	Estimates of effect sizes (e.g. Cohen's *d*, Pearson's *r*), indicating how they were calculated

Our web collection on statistics for biologists contains articles on many of the points above.

Software and code

Policy information about availability of computer code

Data collection	The RNA sequencing was performed using illumina HiSeq2500 platform (Functional Genomics Center, Zurich, Switzerland).
Data analysis	Geneious (6.1.7)<br>SOAPdenovo2 (r240)<br>KmerGenie (1.7039)<br>R (4.3.0)<br>Bowtie (2.3.2)<br>Integrated Genome Viewer (2.3.35)<br>BWA (0.7.12)<br>Trinity (2.2.0)<br>Bio-Rad Laboratories Image Lab (4.1)<br>ImageJ (2.15.0)

For manuscripts utilizing custom algorithms or software that are central to the research but not yet described in published literature, software must be made available to editors and reviewers. We strongly encourage code deposition in a community repository (e.g. GitHub). See the Nature Portfolio guidelines for submitting code & software for further information.

Data

Policy information about availability of data

All manuscripts must include a data availability statement. This statement should provide the following information, where applicable:

- Accession codes, unique identifiers, or web links for publicly available datasets
- A description of any restrictions on data availability
- For clinical datasets or third party data, please ensure that the statement adheres to our policy

Sequence data from this article is available in Genbank and INSDC database. The source data underlying figures are provided as Supplementary data or Source Data file.

Research involving human participants, their data, or biological material

Policy information about studies with human participants or human data. See also policy information about sex, gender (identity/presentation), and sexual orientation and race, ethnicity and racism.

Reporting on sex and gender	N/A
Reporting on race, ethnicity, or other socially relevant groupings	N/A
Population characteristics	N/A
Recruitment	N/A
Ethics oversight	N/A

Note that full information on the approval of the study protocol must also be provided in the manuscript.

Field-specific reporting

Please select the one below that is the best fit for your research. If you are not sure, read the appropriate sections before making your selection.

☒ Life sciences ☐ Behavioural & social sciences ☐ Ecological, evolutionary & environmental sciences

For a reference copy of the document with all sections, see nature.com/documents/nr-reporting-summary-flat.pdf

Life sciences study design

All studies must disclose on these points even when the disclosure is negative.

Sample size	Sample size was determined based on experimental trials and with consideration of previous publication on similar experiments to allow for confident statistical tests. No statistical methods were used to predetermine sample sizes. Length of silique and number of seed per silique of each genotype were measured 5 times (see Source Data). This number is sufficient considering most studies use three independent crosses for such experiments. Exact P-values are shown in Supplementary Table 7 as described in the Statistics and reproducibility section in Methods.
Data exclusions	No data was excluded from the analysis.
Replication	Crossing experiments were conducted at least 4 times (see Supplementary Information for exact numbers). As described in Statistics and reproducible section in Methods, the results of all RT-PCR gel images were verified by two more independent experiments. Three replicates were conducted for qPCR. All experiments were reproducible.
Randomization	Plant location in growth chambers was randomized.
Blinding	Since we properly randomized the plants in the growth chamber, we think that blinding is not required in such a case.

Reporting for specific materials, systems and methods

We require information from authors about some types of materials, experimental systems and methods used in many studies. Here, indicate whether each material, system or method listed is relevant to your study. If you are not sure if a list item applies to your research, read the appropriate section before selecting a response.

Materials & experimental systems

n/a	Involved in the study
[x]	[ ] Antibodies
[x]	[ ] Eukaryotic cell lines
[x]	[ ] Palaeontology and archaeology
[x]	[ ] Animals and other organisms
[x]	[ ] Clinical data
[x]	[ ] Dual use research of concern
[ ]	[x] Plants

Methods

n/a	Involved in the study
[ ]	[x] ChIP-seq
[x]	[ ] Flow cytometry
[x]	[ ] MRI-based neuroimaging

Dual use research of concern

Policy information about dual use research of concern

Hazards

Could the accidental, deliberate or reckless misuse of agents or technologies generated in the work, or the application of information presented in the manuscript, pose a threat to:

No	Yes
[x]	[ ] Public health
[x]	[ ] National security
[x]	[ ] Crops and/or livestock
[x]	[ ] Ecosystems
[x]	[ ] Any other significant area

Experiments of concern

Does the work involve any of these experiments of concern:

No	Yes
[x]	[ ] Demonstrate how to render a vaccine ineffective
[x]	[ ] Confer resistance to therapeutically useful antibiotics or antiviral agents
[x]	[ ] Enhance the virulence of a pathogen or render a nonpathogen virulent
[x]	[ ] Increase transmissibility of a pathogen
[x]	[ ] Alter the host range of a pathogen
[x]	[ ] Enable evasion of diagnostic/detection modalities
[x]	[ ] Enable the weaponization of a biological agent or toxin
[x]	[ ] Any other potentially harmful combination of experiments and agents

Plants

Seed stocks	All plant samples were reported by previous studies (see Materials and Methods, Supplementary Table 8)
Novel plant genotypes	Agrobacterium-mediated floral dip transformation was conducted. Four or more independent lines in T2 generations were analyzed.
Authentication	No new seed stocks were obtained. Focal phenotypes were observed by independent transgenic lines, strongly suggesting that they were not caused by secondary effects such as second site T-DNA insertions.

ChIP-seq

Data deposition

- [ ] Confirm that both raw and final processed data have been deposited in a public database such as GEO.
- [ ] Confirm that you have deposited or provided access to graph files (e.g. BED files) for the called peaks.

Data access links<br>*May remain private before publication.*	*For "Initial submission" or "Revised version" documents, provide reviewer access links. For your "Final submission" document, provide a link to the deposited data.*

Files in database submission | *Provide a list of all files available in the database submission.*

Genome browser session (e.g. UCSC) | *Provide a link to an anonymized genome browser session for "Initial submission" and "Revised version" documents only, to enable peer review. Write "no longer applicable" for "Final submission" documents.*

Methodology

Replicates | *Describe the experimental replicates, specifying number, type and replicate agreement.*

Sequencing depth | *Describe the sequencing depth for each experiment, providing the total number of reads, uniquely mapped reads, length of reads and whether they were paired- or single-end.*

Antibodies | *Describe the antibodies used for the ChIP-seq experiments; as applicable, provide supplier name, catalog number, clone name, and lot number.*

Peak calling parameters | *Specify the command line program and parameters used for read mapping and peak calling, including the ChIP, control and index files used.*

Data quality | *Describe the methods used to ensure data quality in full detail, including how many peaks are at FDR 5% and above 5-fold enrichment.*

Software | *Describe the software used to collect and analyze the ChIP-seq data. For custom code that has been deposited into a community repository, provide accession details.*
