# Supplementary material for: Dominance in self-compatibility between subgenomes of allopolyploid Arabidopsis kamchatica shown by transgenic restoration of self-incompatibility
Source: Nat Commun. 2023 Nov 29;14:7618. doi: 10.1038/s41467-023-43275-2 (PMC10687001; doi:10.1038/s41467-023-43275-2)
Supplement: Supplementary file 4 — Source Data [file 41467_2023_43275_MOESM4_ESM.zip › Source Data of uncropped gel images.pdf]

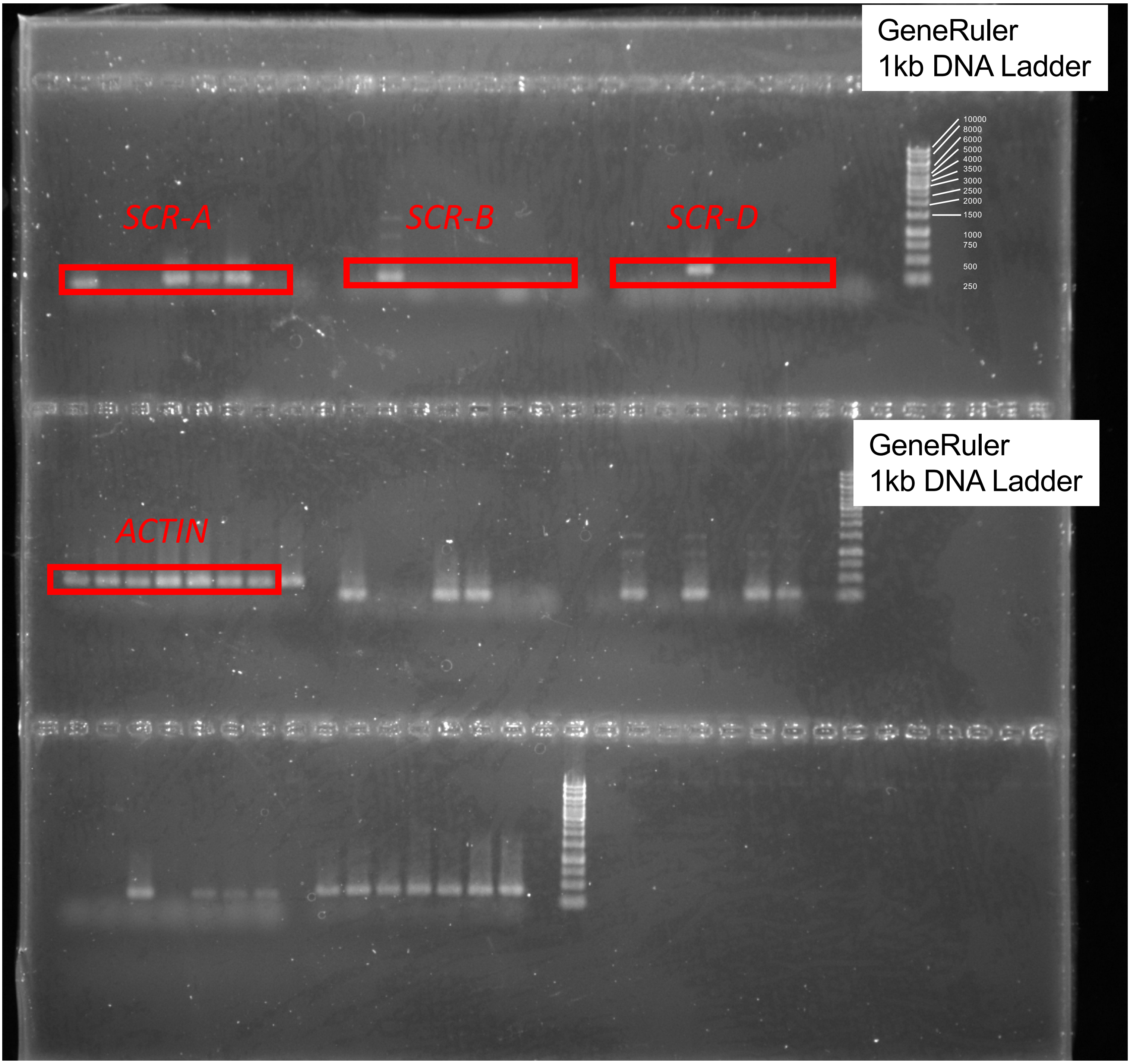

Figure 1c original gel image

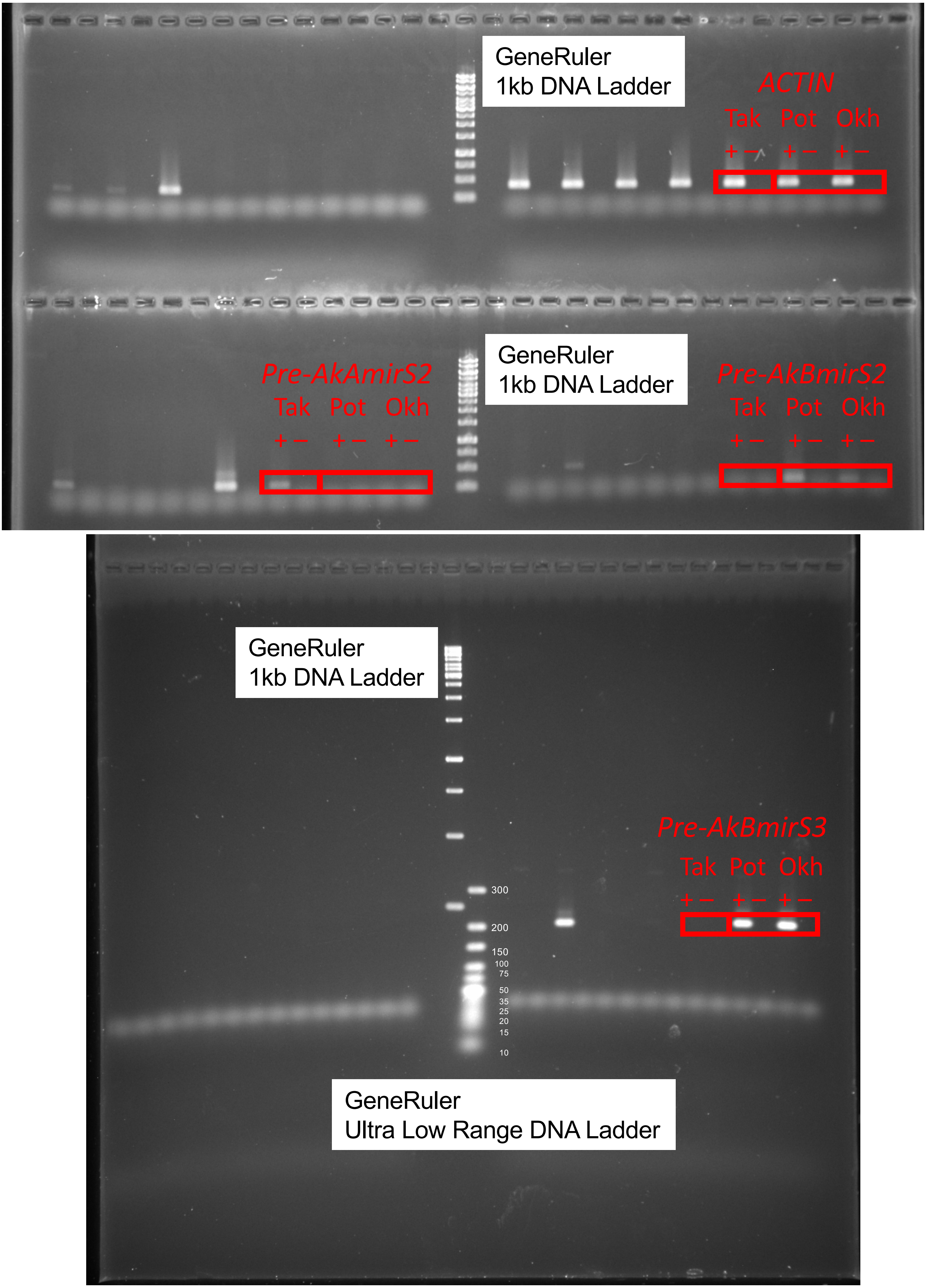

Figure 1d original gel image

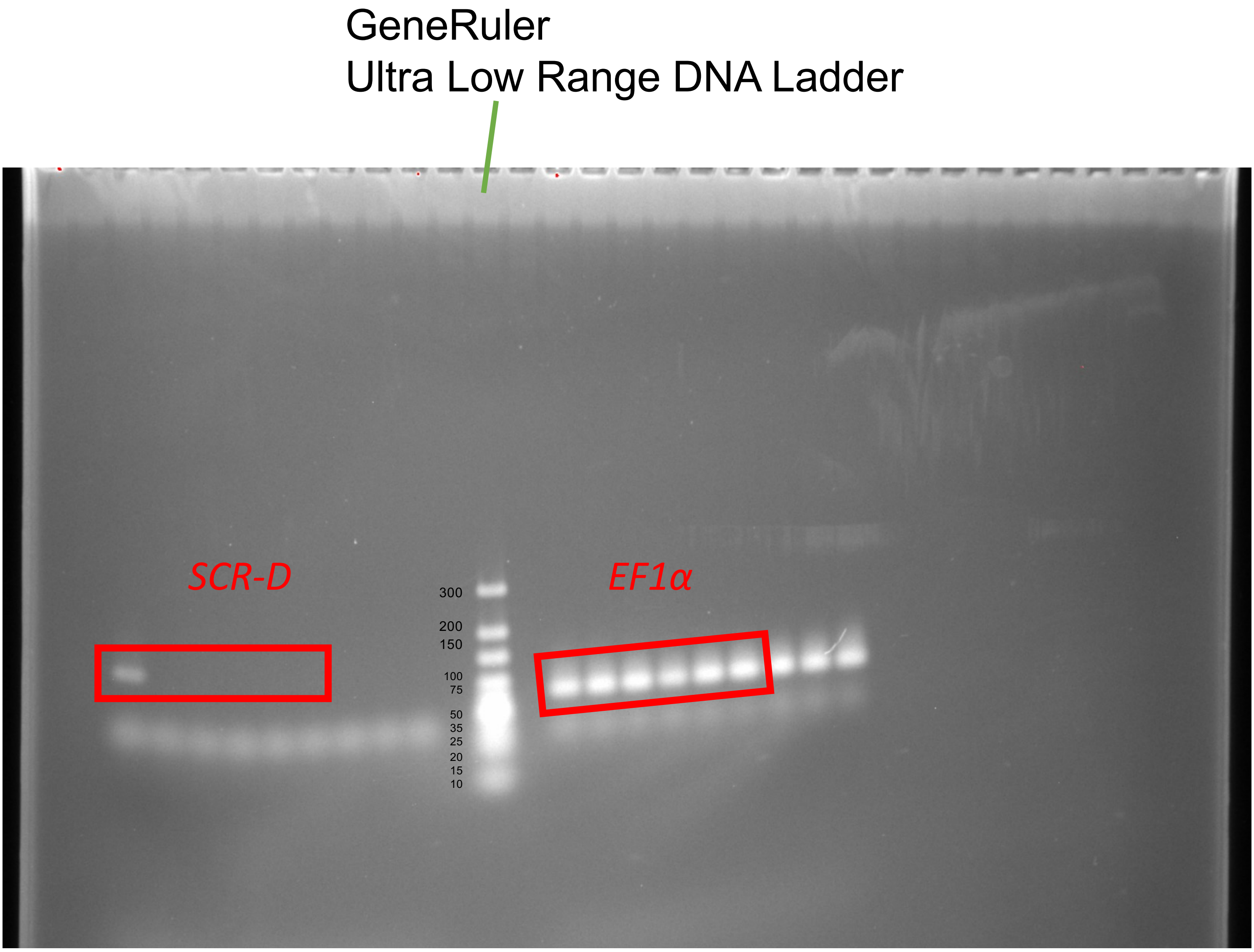

Figure 3a original gel image

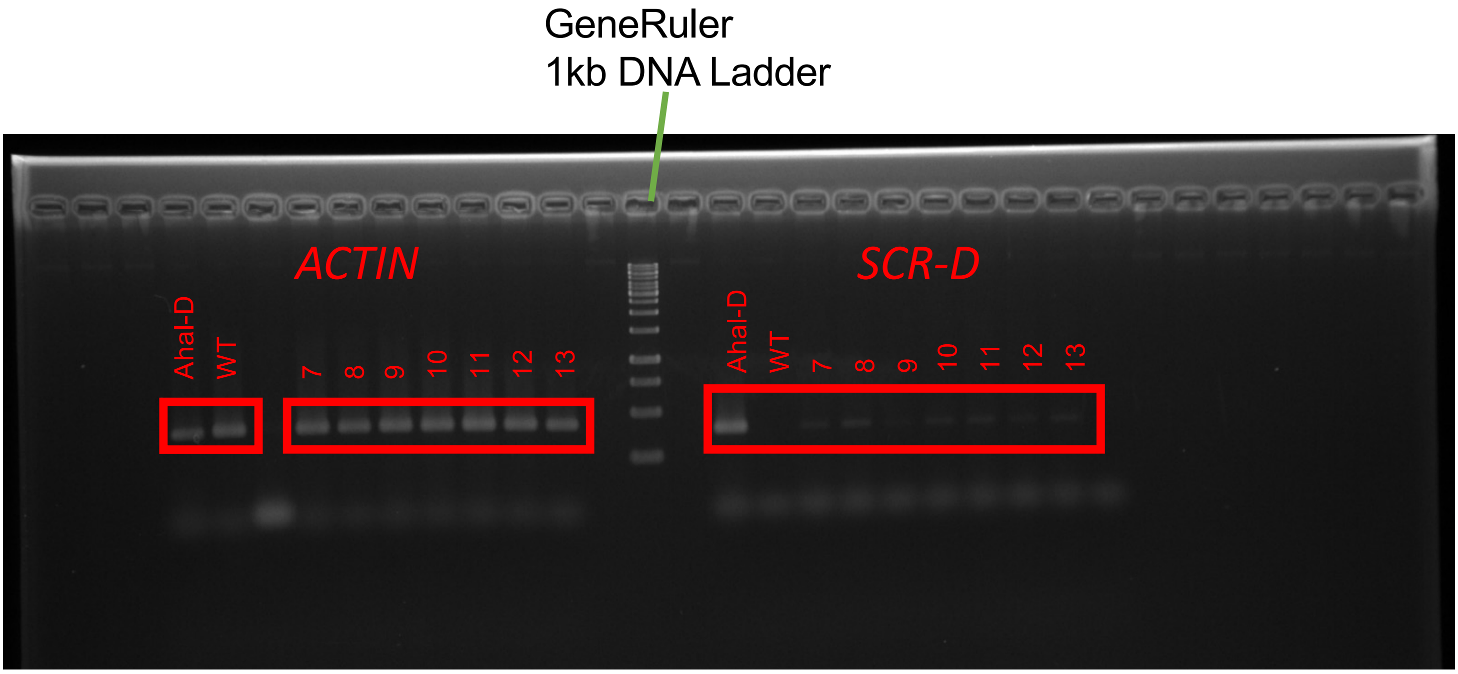

Figure 4a original gel image

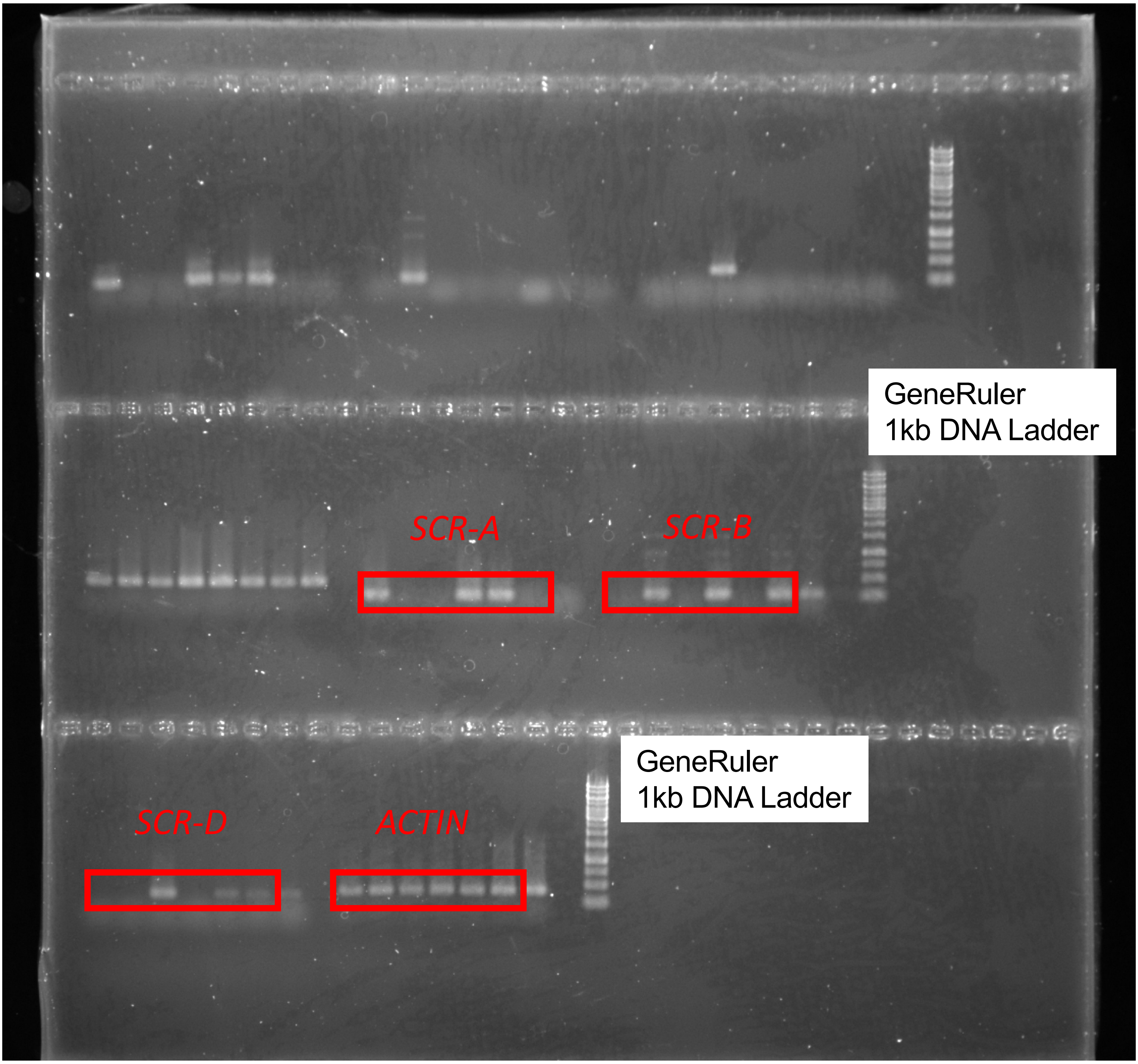

Supplementary Fig. 1b original gel image

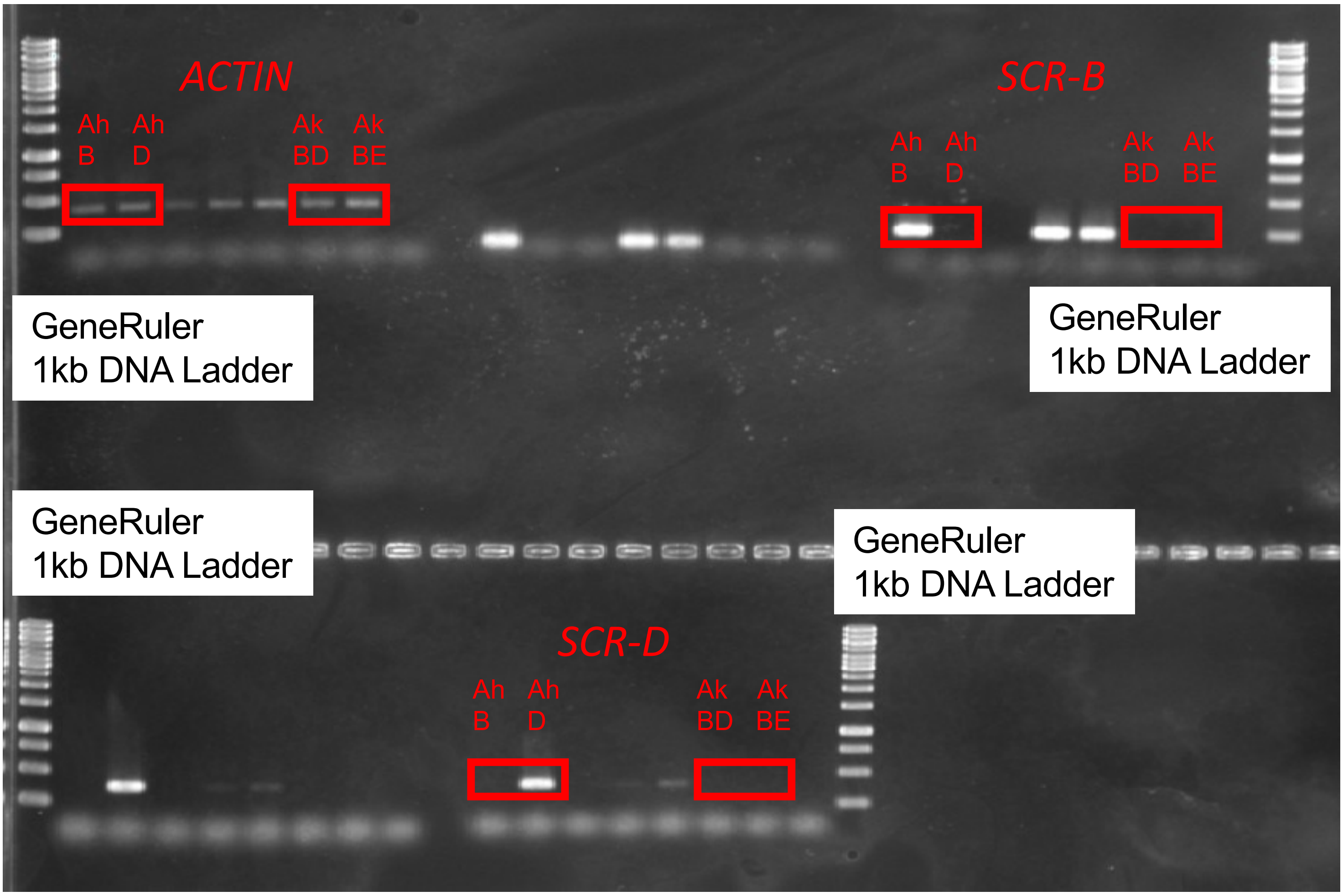

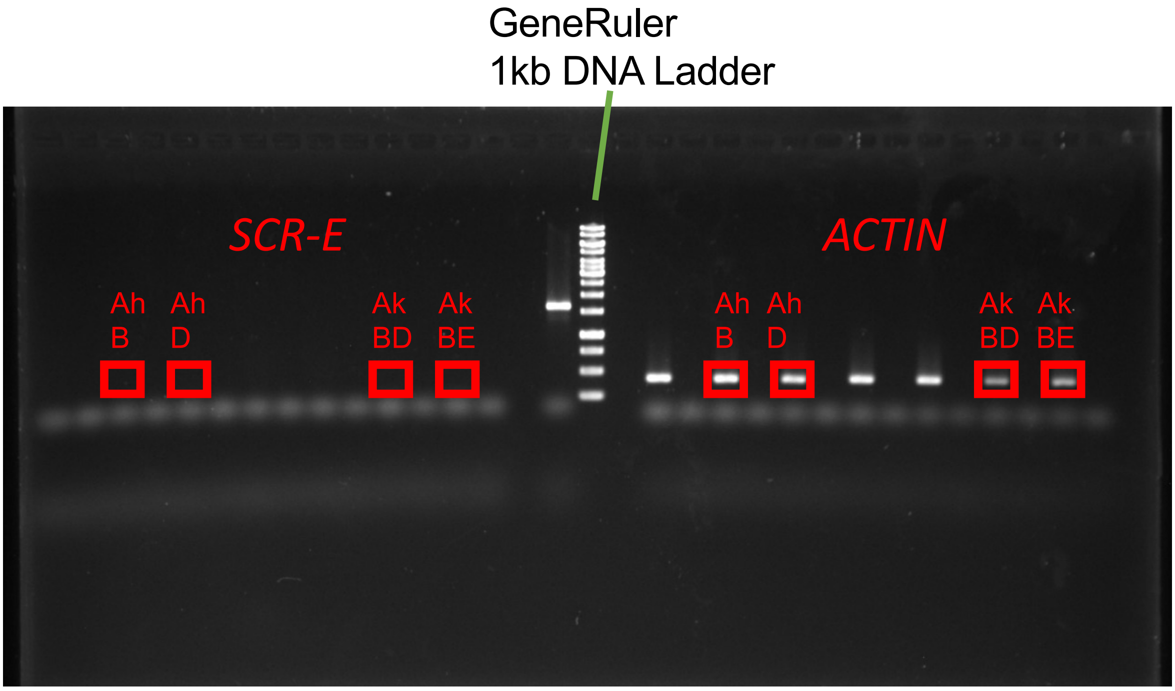

Supplementary Fig. 1c original gel image
ACTIN was run on the both gels and the one above was used in the Supplelentary Fig. 1c.

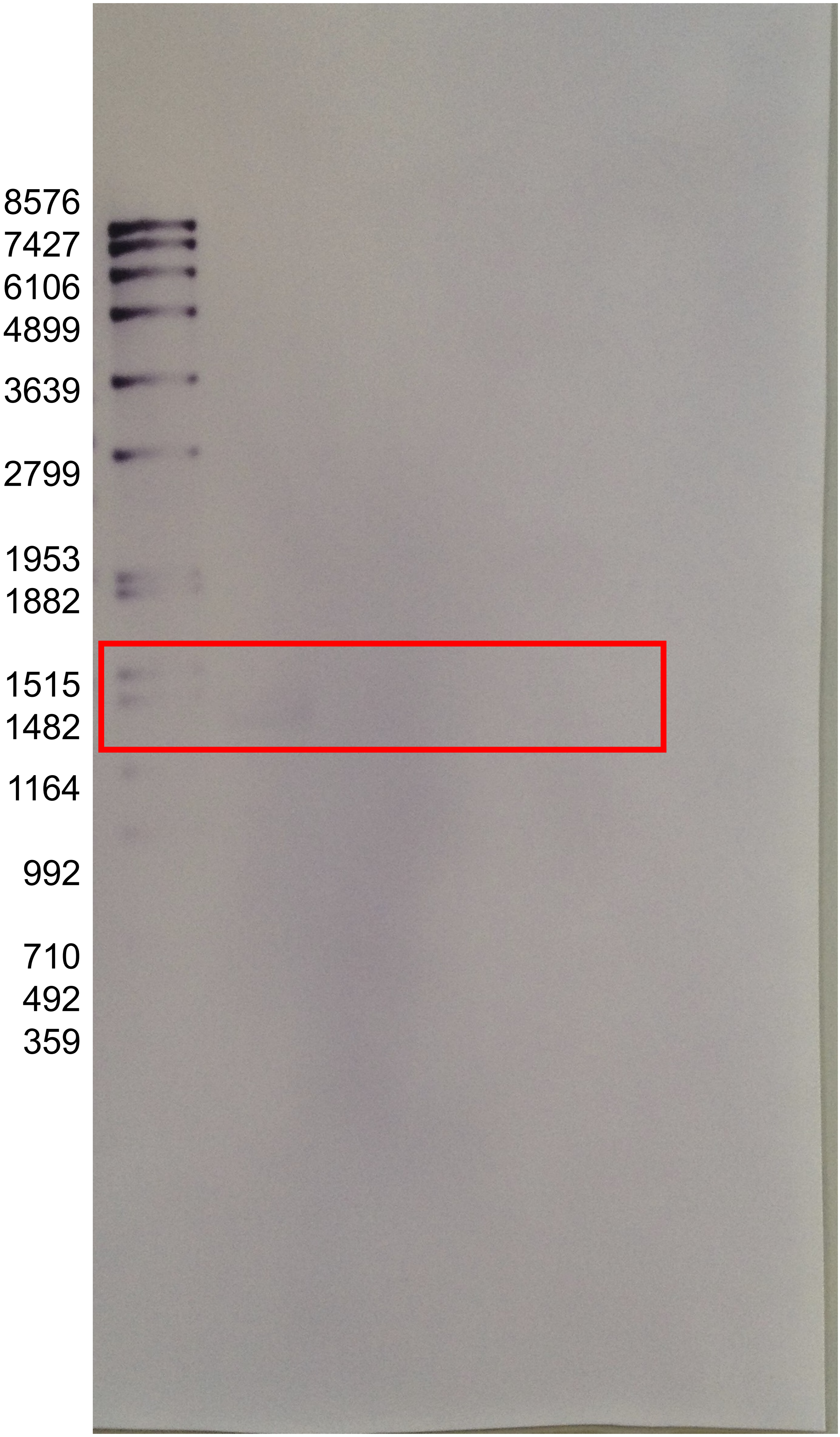

Supplementary Fig. 4a original Southern blotting image
